# TRAF6 promotes osteogenesis in ADSCs through Raf-Erk-Merk-Hif1-a pathway

**DOI:** 10.1080/21623945.2023.2193280

**Published:** 2023-04-02

**Authors:** Xiang-Dong Liu, Jian Zheng, Shuang Song, Zi-Jun Chen, Yu-Xi Wang, Si-Jia Zhang, Guan-Hua Zhang, Qun Lu, Ying-Liang Song

**Affiliations:** a State Key Laboratory of Military Stomatology and National Clinical Research Center for Oral Diseases and Shaanxi Engineering Research Center for Dental Materials and Advanced Manufacture, Department of Implant Dentistry, School of Stomatology, The Fourth Military Medical University, Xi’an, P.R. China; b Department of Implant Dentistry, College of Stomatology, Xi’an Jiaotong University, Xi’an, China; c State Key Laboratory of Military Stomatology and National Clinical Research Center for Oral Diseases and Shaanxi Key Laboratory of Oral Diseases, Department of Operative Dentistry and Endodontics, School of Stomatology, The Fourth Military Medical University, Xi’an, P.R. China

**Keywords:** ADSCs, TRAF6, Hif1a, Osteogenesis

## Abstract

Critical-size defects (CSDs) are challenging oral clinical issues that need to be solved. Adipose-derived mesenchymal stem cells (ADSCs) and gene therapy offer a new target to solve these issues. Consequently, ADSCs attract more and more attention because of advantages such as easy obtainability and no ethical concerns. TNF receptor-associated factor 6 (TRAF6) is a significant binding protein both of tumour necrosis factor superfamily and of the toll/interleukin-1 receptor superfamily. Evidence is accumulating that TRAF6 inhibited osteoclast formation and promoted the proliferation of multiple myeloma cell lines and bone resorption. Here, we reported that overexpression of TRAF6 enhanced the proliferation, migration and osteogenesis of ADSCs through Raf-Erk-Merk-Hif1a pathway. Cell sheet of ADSCs combined with TRAF6 accelerated the healing of CSDs. In a word, TRAF6 enhanced osteogenesis, migration and proliferation through Raf-Erk-Merk-Hif1a pathway.

## 1. Introduction

Bone loss represents a challenging oral clinical issue, especially in large defects such as critical-size defects (CSDs), where surgical intervention is needed because of its lack of self-regeneration [1]. To facilitate CSD healing, regenerative medicine and gene therapy are widely applied nowadays [2].

Human mesenchymal stem cell (hMSC) is a kind of self-renewing multipotent cell derived from different tissues, including bone marrow, adipose tissue, blood, cord, pulp and periodontal ligaments [3,4]. hMSCs could differentiate into any types of cells or organism and be able to self-renew [5,6]. Bone-marrow stromal cells (BMSCs) are obtained from adult bone with a pluripotent differentiation and can usually be used in bone tissue engineering [7]. In contrast to BMSCs, adipose-derived mesenchymal stem cells (ADSCs) have several advantages such as easy obtainability, abundant sources, low technique sensitivity and no ethical issues [8]. However, low osteogenic differentiation is an obstacle to applying ADSCs to bone tissue engineering [9]. Many efforts have been made to improve the osteogenic tendency, including gene therapy [10–12].

Tumor necrosis factor (TNF) receptor-associated factor 6 (TRAF6) is a ubiquitin ligase which is a significant binding protein of both TNF superfamily and toll/IL-1 receptor (TIR) superfamily [13,14]. It was first recognized as the initiation of innate immunity and adaptive immune responses induced by the recognition of different molecular pathogens [14,15]. C-terminal domain and N-terminal domain were special domains of TRAF6 with its functions as an E3 ubiquitin ligase by integrating with various kinases and regulating signalling pathways [16]. TRAF6 acts as the intersection of the activation of the NF-κB signalling pathway and the mitogen-activated protein kinase signalling pathway [17]. Osteoclasts are defective in TRAF6-deficient mice [18]. Interferon-γ-induced TRAF6 degradation also inhibited osteoclast formation [19]. TRAF6 silencing inhibits the proliferation of MM in cell lines and primary cells but inhibits the formation of osteoclasts and bone resorption [20]. However, whether TRAF6 influenced osteogenesis of ADSCs remains unknown.

In our study, we hypothesized that TRAF6 influenced the osteogenesis of ADSCs and bone formation through Raf-Erk-Mek-hif-1a pathway.

## 2. Results

### 2.1. Identification of ADSCs

**Figure 1.**
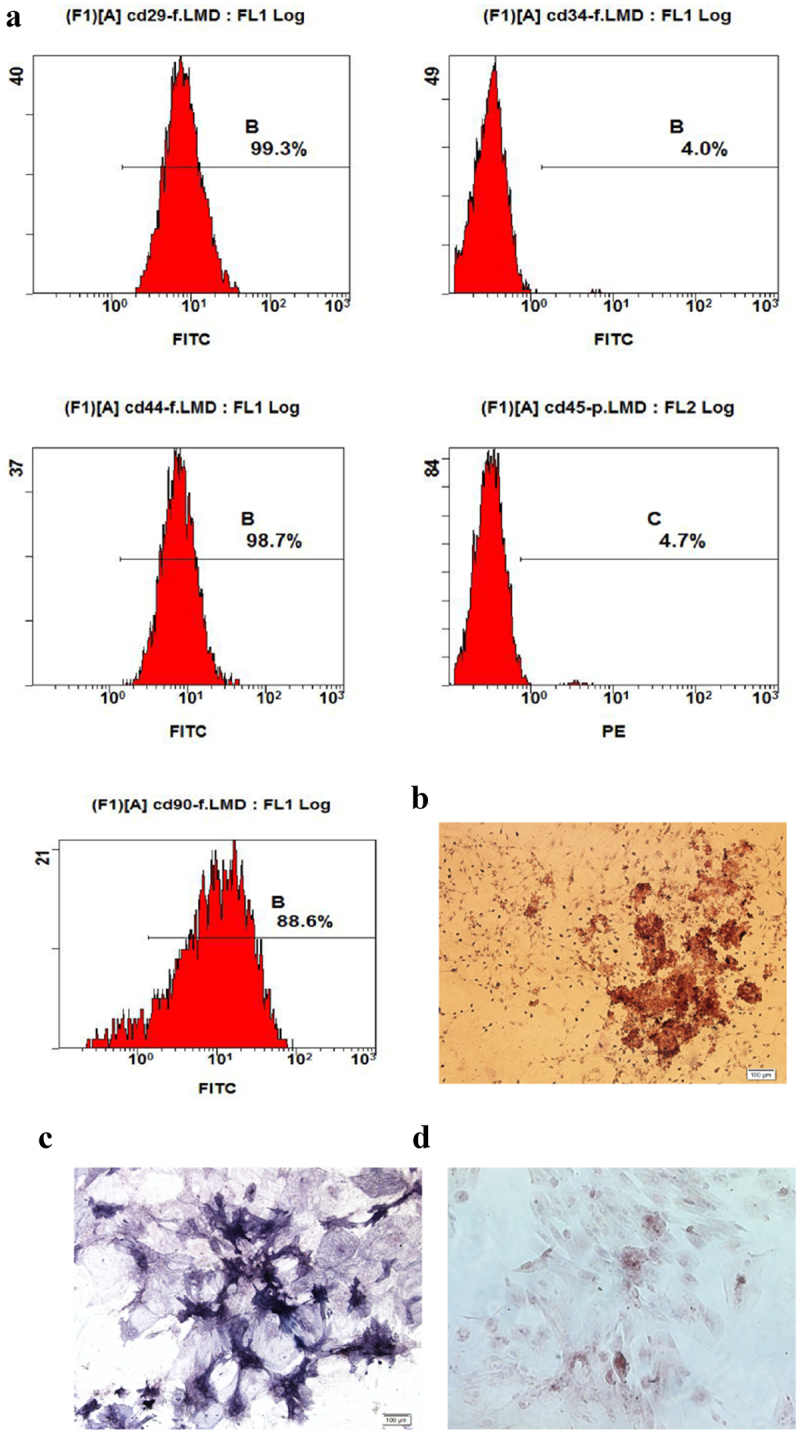
Characteristics of ADSCs. a: The special surface markers including CD29, CD34, CD44, CD45 and CD90 were detected by flow cytometry analysis. b: Osteogenesis was detected by alizarin red stanning. c: Osteogenesis was detected by ALP stanning. d: Adipogenesis was detected by oil red stanning.

Flow cytometry and multi-lineage potential were used to identify the characteristics of ADSCs. As shown in Figure 1a, the expression of CD29, CD44 and CD90 was positive, while the expression of CD34 and CD45 was negative. Furthermore, it has been confirmed that the cells differentiate osteoblasts (Figure 1b and c) and adipocytes (Figure 1d).

### 2.2. Concentration of TRAF6 on ADSCs

**Figure 2.**
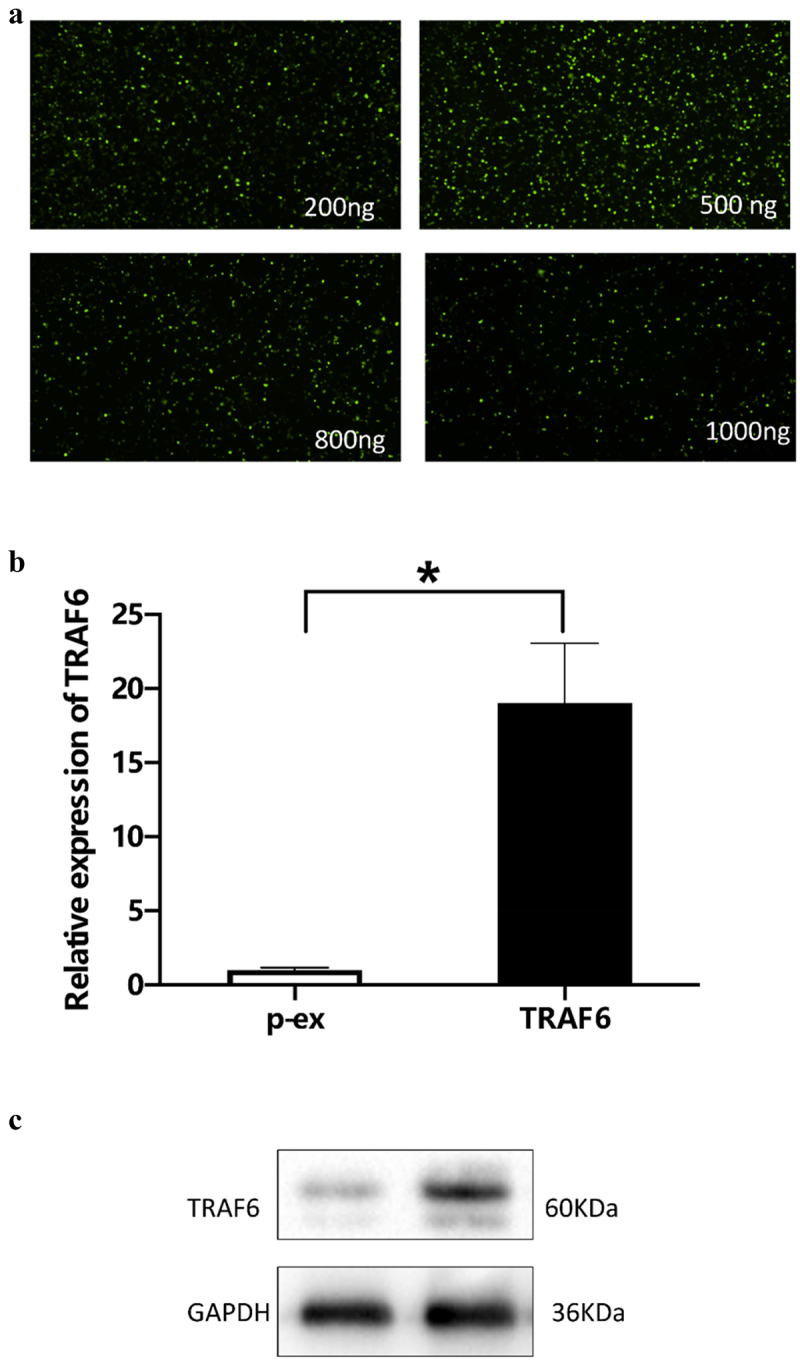
Verification of concentration of TRAF6 vector. a: 500 ng vector of TRAF6 was detected as an optimum concentration by immunofluorescence. b: Transfection efficacy was detected by qRT-PCR. c: Transfection efficacy was detected by Western blot. A t-test was used to compare p-ex and TRAF6. Data are expressed as mean ± SD referred to the control (*p<0.05) P-EX: blank vector; TRAF6: vector of overexpression of TRAF6.

To detect the effect of TRAF6 on ADSCs, overexpression of TRAF6 was cloned from Sangon Company (Sangon, Shanghai). Immunofluorescence method was used to verify the optimum concentration, and the result showed that 500 ng was an optimum concentration (Figure 2a). Quantitative real-time polymerase chain reaction (qRT-PCR) and Western blot elucidated that TRAF6 successfully transfected into ADSCs and prolonged the RNA (Figure 2b) and protein level (Figure 2c).

### 2.3. Effects of TRAF6 on ADSCs

**Figure 3.**
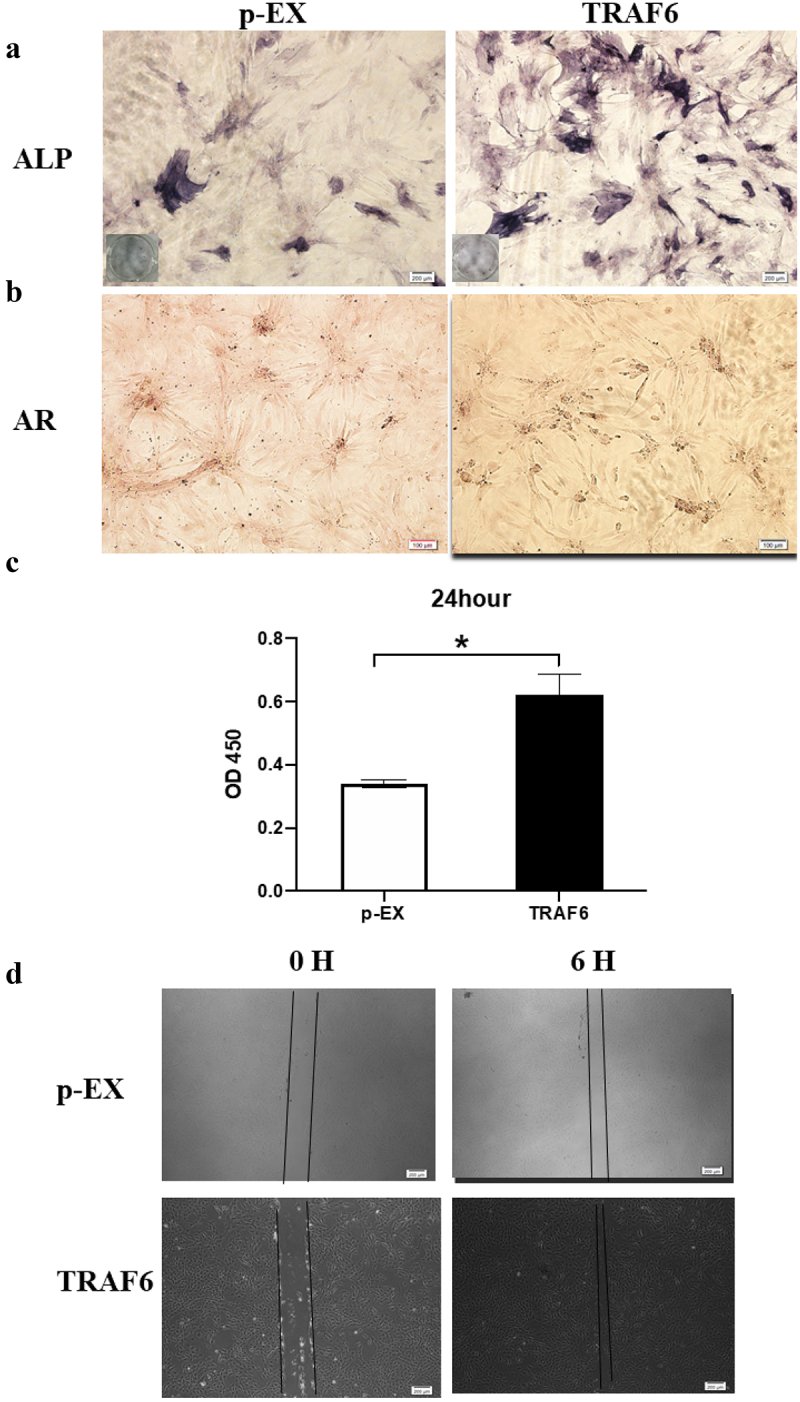
The effects of TRAF6 on proliferation, migration and osteogenesis of ADSCs. a: The osteogenesis detected by ALP. b: The osteogenesis detected by AR. c: The proliferation rate analysed by CCK-8 after transfection for 24 h. d: The migration rate analysed by wound healing. A t-test was used to compare p-ex and TRAF6. Data are expressed as mean ± SD referred to the control (*p<0.05); P-EX: blank vector; TRAF6: vector of overexpression of TRAF6, ALP: alkaline phosphatase, AR: alizarin red.

TRAF6 promoted osteogenesis detection by alkaline phosphatase (ALP) and Alizarin red stanning (Figure 3a and b). CCK-8 results showed that TRAF6 enhanced proliferation on ADSCs after transfecting for 24 h (Figure 3c). As shown in Figure 3d, TRAF6 accelerated the migration through wound healing.

### 2.4. Mechanisms of TRAF6 on ADSCs

**Figure 4.**
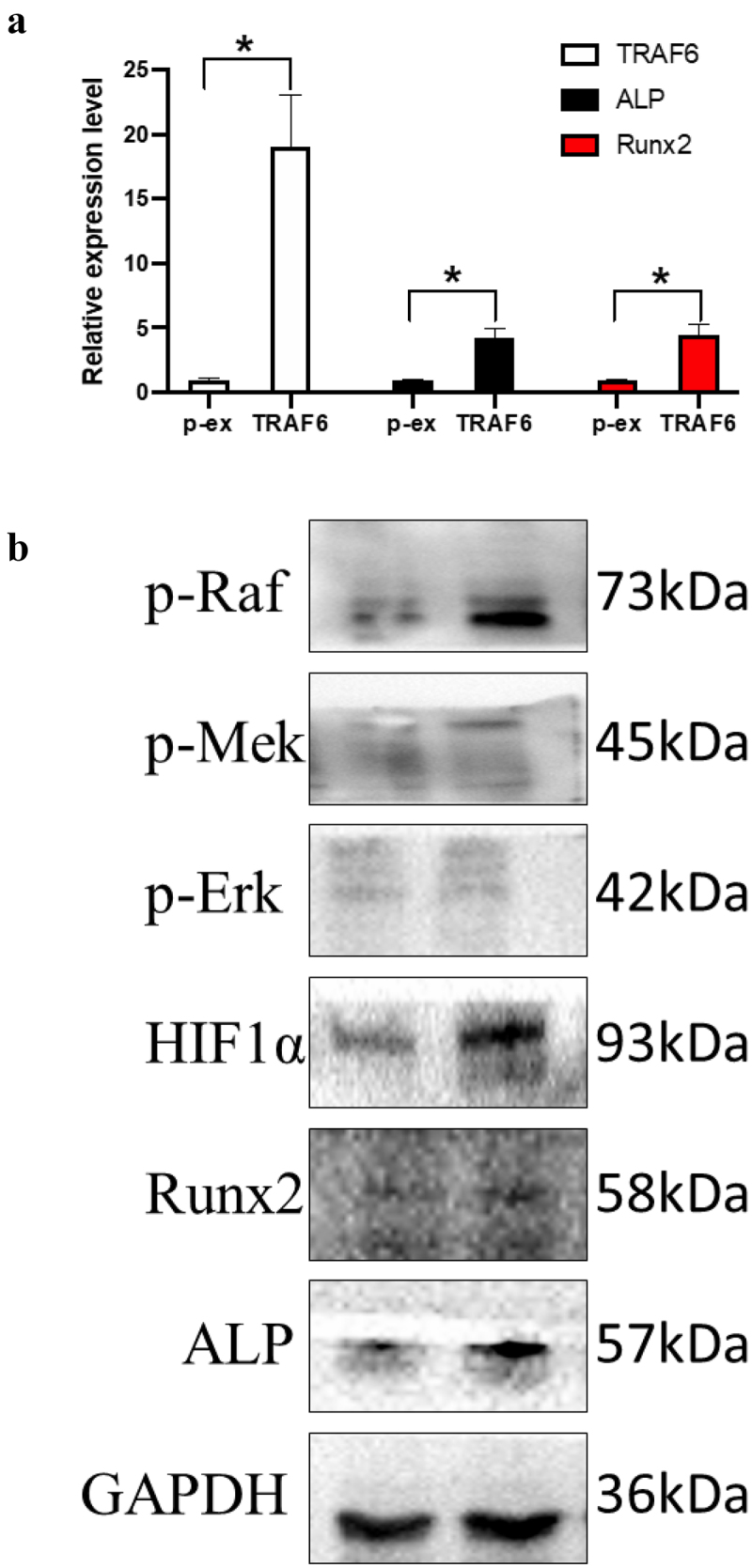
The mechanism of TRAF6 promoting osteogenic differentiation. a: The mRNA expression level of ALP, RUNX2 and TRAF6 was detected by qRT-PCR. b: The protein expression level of HIF1a, ALP, RUNX2 and TRAF6 was detected by Western blot. A t-test was used to compare p-ex and TRAF6. Data are expressed as mean ± SD referred to the control (*p<0.05) P-EX: blank vector; TRAF6: vector of overexpression of TRAF6.

TRAF6 promoted the RNA expression of TRAF6, ALP and Runx2 (Figure 4a); meanwhile, it promoted the protein level of Runx2 and ALP. TRAF6 also overexpressed the protein level of p-Raf, p-Mek, p-Erk and Hif-1a, which means TRAF6 could activate the Raf-Mek-Erk-Hif-1a pathway (Figure 4b).

### 2.5. Osteogenesis effect of TRAF6 in vivo

**Figure 5.**
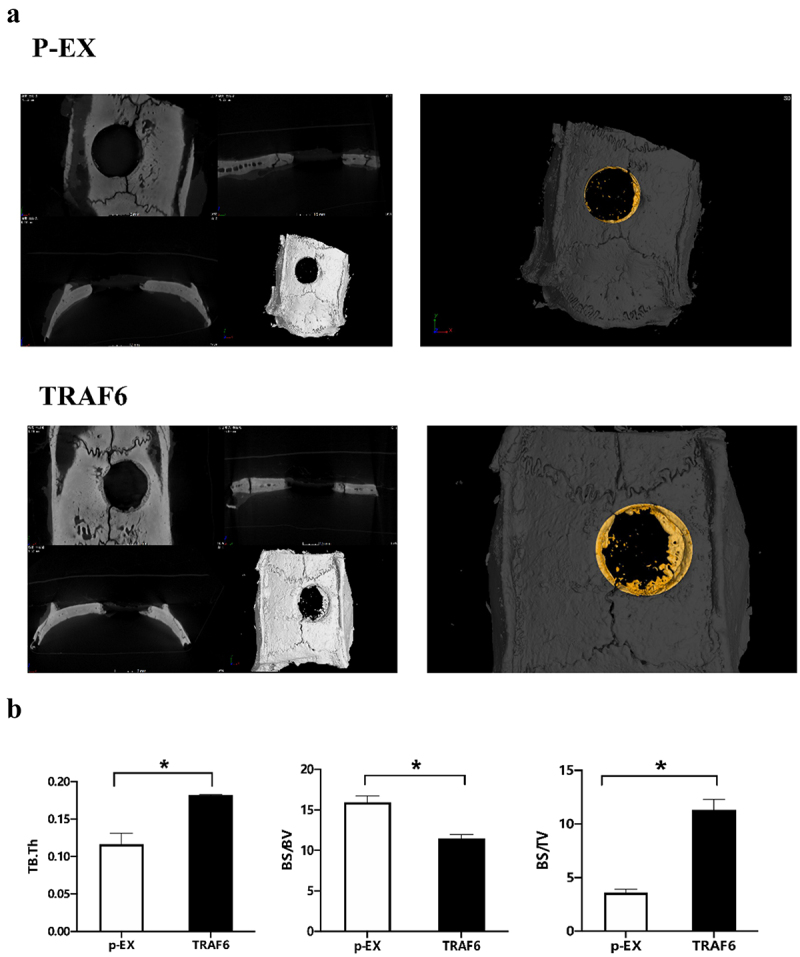
The effect of TRAF6 on osteogenesis of ADSCs in vitro. a: The healing of bone defect on ADSCs detected by micro-CT. b: The osteointegration parameters detected by micro-CT. A t-test was used to compare p-ex and TRAF6. Data are expressed as mean ± SD referred to the control (*p<0.05) P-EX: blank vector; TRAF6: vector of overexpression of TRAF6.

Bone defect was exhibited and filled with cell membrane after transfecting TRAF6. TRAF6 promoted the healing of bone defect (Figure 5a) and bone volume/tissue volume (BV/TV) and bone surface area tissue volume ratio (BS/TV), while inhibited BS/BV (Figure 5b).

### 2.6. Histological observations and analysis

**Figure 6.**
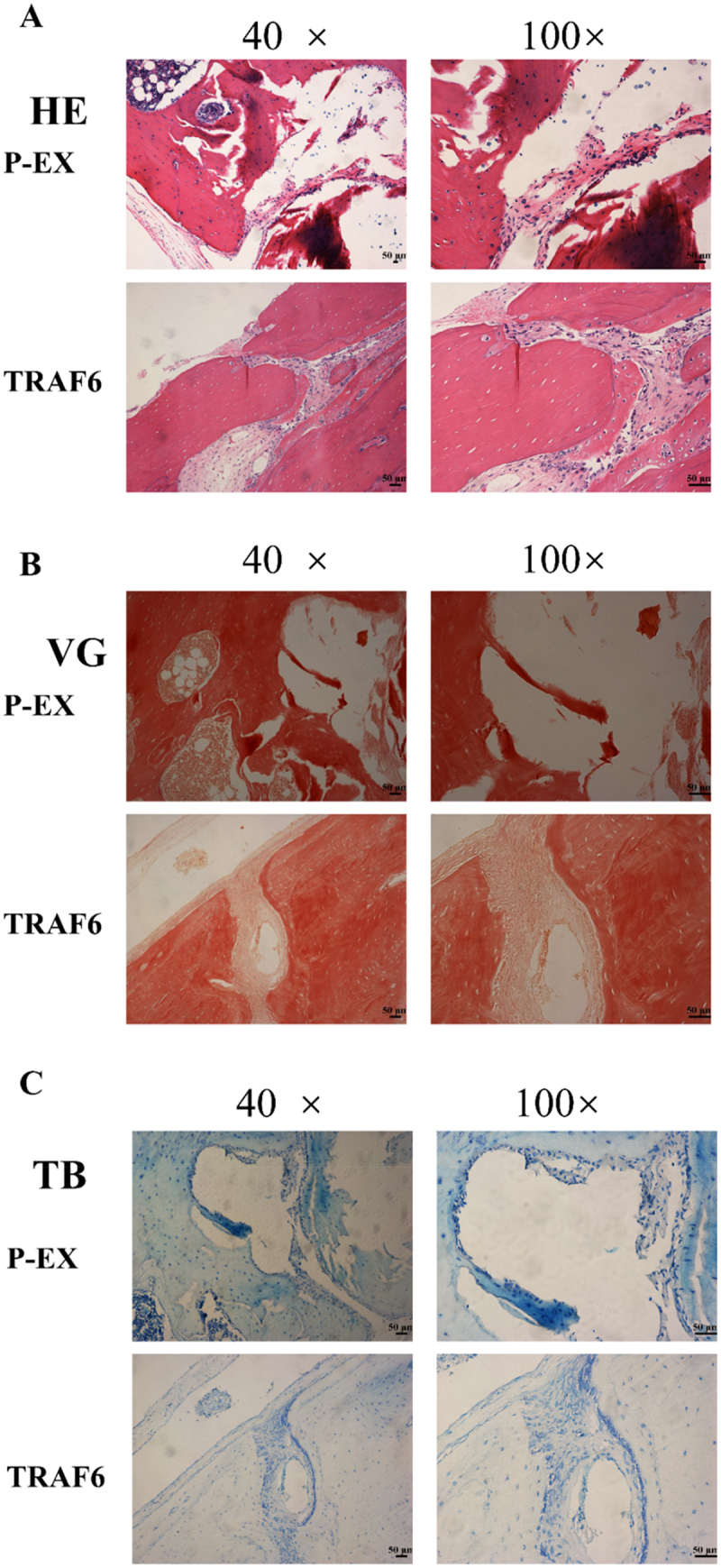
The osteogenic effect of TRAF6 was analysed by histomorphology staining. a: The osteogenic effect of TRAF6 was analysed by HE stanning. b: The osteogenic effect of TRAF6 was analysed by VG staining. c: The osteogenic effect of TRAF6 was analysed by TB staining. P-EX: blank vector; TRAF6: vector of overexpression of TRAF6. HE: Hematoxylin and eosin; VG: Van Gieson; TB: Toluidine blue.

Haematoxylin and eosin (HE) stanning showed a higher new bone formation in the TRAF6 overexpression group (Figure 6a). Results of Van Gieson (VG) stanning declared a stained darker and increased in collagen deposition bone tissue in the TRAF6 overexpression group (Figure 6b). Results of Toluidine blue (TB) stanning dedicated that a much new bone formation in the TRAF6 overexpression group (Figure 6c).

### 2.7. A diagram of all procedures

**Figure 7.**
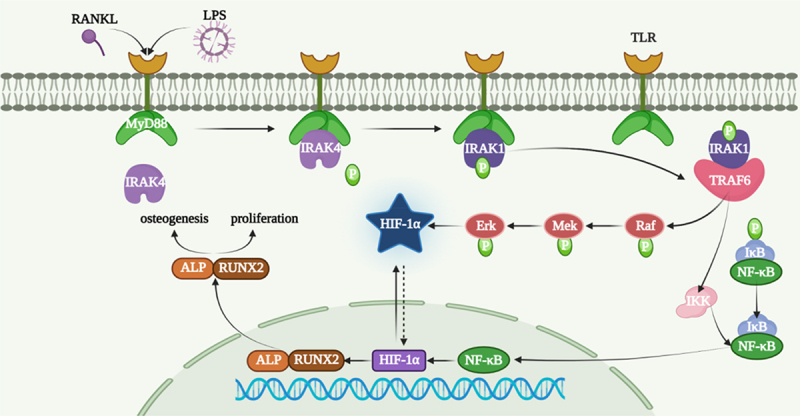
A diagram of the procedure of Raf-Mek-Erk-Hif-1a pathway.

A diagram of the procedure of Raf-Mek-Erk-Hif-1a pathway is shown in Figure 7.

## 3. Discussion

In our study, we found that overexpression of TRAF6 promoted osteogenesis, proliferation and migration of ADSCs through Raf-Mek-Erk-Hif-1a signal pathway. Meanwhile, TRAF6 induced the new bone formation in a CDS model of rat through ADSC cell sheet.

In fact, CSDs, which are usually resulting from bone tumours, bone diseases and injuries, will not come into being the new bone of the patients or animal models completely. At the same time, new bone formation is a complex process involving mesenchymal stem cells (MSC) and a variety of biological factors [21,22]. Professor Nilveus has developed and described models of critical-size supra-alveolar periodontal defects [23] and subsequent models of critical-size supra-alveolar perialveolar defects [24] for the evaluation of periodontal and alveolar bone regeneration techniques. ADSCs were defined as a potential seed cell for regenerative medicine because of their easy accessibility and no ethics properties [25,26]. Although there are obstacles to improving the osteogenic differentiation of ADSCs, some efforts have been made to improve the osteogenesis of ADSCs. For instance, Zhang et al. found that circRNA-vgll3/miR-326-5p/integrin α5 (Itga5) pathway promoted the osteogenesis of ADSCs [27]. Liu et al. showed a much more osteogenic differentiation tendency of miR-145-5p inhibitors on ADSCs [28]. GLP-1 enhanced osteoblastic mineralization through the higher expression of OCN, Runx2 and collagen I [29]. Receptor activator of NF-kB ligan (RANKL) is the most important cytokine for inducing osteoclast differentiation and affecting its survival, maturation and function [30]. The binding of RANKL to RANK occurs after the recruitment of tTRAFs besides TRAF6 [31]. TRAF6 is a ubiquitin ligase binding both TNF superfamily and TIR superfamily. The vital roles of TRAF6 in bone growth and bone remodelling were determined by osteoclast differentiation and bone resorption pit assays [32,33]. TRAF6 plays a vital role in NF-KB signalling pathway and is an upstream of LPS-induced osteoclastogenesis [34,35]. The bone loss that occurs in periodontitis depends on RANKL produced by osteoblasts and periodontal ligament cells [36]. Meanwhile, some scholars have found that TRAF6 was upregulated during osteoclastogenesis after LPS-inducing osteoclast [35,37]. SiRNA of TRAF6 inhibited the proliferation and promoted the apoptosis on myeloma and multiple myeloma (MM) cells [38]. HIF-1α is a critical factor for bone formation [39]. Osteoblasts stayed on the surface of new bone and sensed a decrease in oxygen levels, and HIF-1α is a vital mediator during this process [40]. As shown in Figure 7, Erk and Mek are upstream of Hif-1a [41], which is in accordance with our results. Our results showed that TRAF6 promoted the protein level of p-Raf, p-Erk, p-Mek and HIF-1α.

In summary, TRAF6 induced osteogenesis, proliferation and migration but inhibited the adipogenesis in vivo and in vitro by overexpression of Raf-Mek-Erk-Hif-1a pathway. In conclusion, TRAF6 promoted the osteogenic differentiation and proliferation and migration on ADSCs through Raf-Mek-Erk-Hif-1a pathway but inhibited adipogenic differentiation. In vivo, TRAF6 in cell sheet could induce new bone formation. All results indicated that TRAF6 could be an inducible factor for bone defect. Our data also offer a clinical application of ADSC and shed new light on the gene therapy of TRAF6 to accelerate CDS wound healing and attenuate new bone formation.

## 4. Materials and methods

### 4.1. Ethics and animals

All animal experiments were supervised and approved by the Animal Ethics Committee of School of Stomatology of the Fourth Military Medical University (kq-011). All procedures were performed according to the ARRIVE guidelines (https://arriveguidelines.org). All methods were in accordance with relevant guidelines including the revised Animals (Scientific Procedures) Act 1986 in the UK and Directive 2010/63/EU in Europe.

### 4.2. Animal model

Twenty-four Sprague–Dawley male rats (250 g ± 10 g) were selected and adaptive fed in a specific pathogen-free condition, which was 21°–23°C temperature, 30–60% relative humidity and 12 h light/dark in the Experimental Animal Center of the School of Stomatology, the Fourth Military Medical University. All rats were randomly divided into two groups (*n* = 12). Model of critical-sized calvaria defect was exhibited as previously described [42] with sterile conditions under anaesthesia. Group of p-ex was transfected with blank vectors and group of TRAF6 which was transfectedinto the overexpression vector of TRAF6. After 4 weeks, rats were sacrificed with overdose of anaesthesia and skulls were selected for micro-CT and HE, VG and TB.

### 4.3. Micro-CT

After implantation for 4 weeks, the new bone in the defective area was reconstructed by the micro-CT system. Using the medium resolution mode, the samples were scanned at a thickness of 0.018 mm of each slice, with 1,024 reconstruction matrix together with a 200 ms integration time. After 3D reconstruction, BMD, BV/TV, Tb.N, Tb.Th and Tb.Sp were automatically determined in order to verify the osteoporotic model, and BMD and BV/TV values in the defect regions were used to evaluate new bone formation by auxiliary software (Scanco Medical, Bassersdorf, Switzerland).

### 4.4. Histomorphology section and stanning

After operation for 4 weeks, bone tissue was fixed with 4% paraformaldehyde overnight, and then embedded in paraffin, and sect continuously at 4 µm. These slices were stained with HE, VG and TG as previously described.

### 4.5. Isolation, culture and characterization of ADSCs

ADSCs were isolated from groin fat of young rats as previously described [43]. Adherent cells were cultured in a α-MEM medium with 10% FBS and 1% penicillin–streptomycin. To identify the characteristics of ADSCs, flow cytometry analysis and multi-lineage potential assay were used according to methods previously reported.

### 4.6. Cell transfection

First, 1 × 10^6^ ADSCs were seeded into a six-well plate. Cells were washed with 1× PBS three times and then transfected with 500 ng p-ex or 500 ng TRAF6 using Lipofectamine 3000. Transfection efficiencies were detected by qRT-PCR and Western blot.

### 4.7. Cell proliferation assay

1 × 10^3^ ADSCs were seeded into a 96-well plate. Cells were washed with 1× PBS three times and then transfected with 500 ng p-ex or 500 ng TRAF6 using Lipofectamine 3000. Ten microlitres of cck-8 regents was added into the plate and then incubated in an incubator for 30 min after 24 h. OD value of 450 nm was measured by microplate reader.

### 4.8. Osteogenic induction, ALP and Alizarin assay

Osteogenic induction medium which included 10% foetal bovine serum, 10 μL 10^−8^ mol/L dexamethasone sodium phosphate, 1 mL 100 U/mL penicillin, 1 mL 100 U/mL streptomycin and 1 mL 0.1 mmol/L L-ascorbic acid phosphate was added into a six-well plate after transfection. After inducing for 7 days, ALP stanning was done as previously described. Alizarin assay was used to detect the calcium deposits after 21 days of induction.

### 4.9. Adipogenic induction and oil red assay

Adipogenic induction medium which included 10% foetal bovine serum, 1 mL 100 U/mL penicillin and streptomycin, 5 μL 10^−8^ mol/L dexamethasone sodium phosphate and 250 µl 0.5 mmol/L IBMX was added into a six-well plate after transfection. After inducing for 7 days, oil red stanning was done as previously described.

### 4.10. Scratch test

A scratch line was made using a 10 µl pipette tip. Then, the pictures were captured by a microscope and the plate was removed from the incubator after transfection. The pictures were captured again by a microscope after transfection for 6 h. The pictures were disposed by ImageJ.

### 4.11. RNA isolation and quantitative real-time PCR

Cells were washed three times by 1× PBS after transfection and then digested by TRIzol. Five-hundred microlitres of trichloromethane was added and centrifuged at 4°C at 12,000 rpm for 15 min. One-hundred microlitres of isopropanol was added after gently absorbing the middle layer and centrifuged at 4°C at 9000 rpm for 10 min. RNA sedimented at the bottom after being washed by 70% ethyl alcohol at 4°C at 9000 rpm for 5 min, centrifuging twice. Concentration of RNA was qualified by NanoDrop and transcripted into cDNA by PrimeScript Master Mix (Takara). Relative expression of genes was detected using TB Green® Premix Ex Taq^TM^ II (Takara) by 2^−ΔΔCt^ method. Primer sequence is shown in Table 1.

**Table 1.** Sequencing of primers.

Gene	Forward sequence	Reverse sequence
ALP	CAACGAGGTCATCTCCGTGATG	TACCAGTTGCGGTTCACCGTGT
Runx2	CCCAGTATGAGAGTAGGTGTCC	GGGTAAGACTGGTCATAGGACC
HIF-1a	TACTCAGCACTTTTAGATGCTGTT	ACGTTCAGAACTTATCCTACCAT
GAPDH	GTCTCCTCTGACTTCAACAGCG	ACCACCCTGTTGCTGTAGCCAA

### 4.12. Western blot

Protein was extracted by RIPA (Takara) and quantified with BCA (bicinchoninic acid). Proteins were separated with sodium dodecyl sulphate–polyacrylamide gel and transferred to PVDF membrane (Millipore, USA). After blocking with 5% skimmed milk, the membranes were incubated overnight with each primary antibody including ALP, RUNX2, TRAF6, p-Raf, p-Erk, p-Mel and HIF1α (Abcam, 1:1000 dilution). Relative secondary antibody (Abcam, 1:5000 dilution) was incubated for 2 h. ECL kit (Solarbio, China) was used to develop the target bands, and the GAPDH was a normalization.

### 4.13. Statistical analysis

GraphPad Prism 7.0 was used for statistical analysis by t-test. Besides, *P* value < 0.05 is considered statistically significant, and all experiments were repeated at least three times.

## Supplementary Material

Supplemental Material

## Data Availability

The data that support the findings of this study are openly available in Adipocyte at URL.

## Abbreviations

ADSCS, adipose-derived stem cells; CSD, critical-size defects; TRAF6, TNF-receptor-associated factor 6; TNF, tumour necrosis factor; TIR, toll/IL-1 receptor; BV/TV, bone volume/tissue volume; BS/TV, bone surface area tissue volume ratio; HE, hematoxylin and eosin; VG, Van Gieson; TB, toluidine blue
