# Supplementary material for: TRAF6 promotes osteogenesis in ADSCs through Raf-Erk-Merk-Hif1-a pathway
Source: Adipocyte. 2023 Apr 2;12(1):2193280. doi: 10.1080/21623945.2023.2193280 (PMC10072064; doi:10.1080/21623945.2023.2193280)
Supplement: Supplemental Material [file KADI_A_2193280_SM3111.docx]

10 20 30 40 50 60


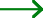
 ATAGCAGAGCTGGTTTAGTGACCGTCAGATCCGCTAGCGCTACCGGACTCAGATCTCGAGGCCACCAT A2506_1-CMV_F-Z171106087A_G01.ab1(50>600)


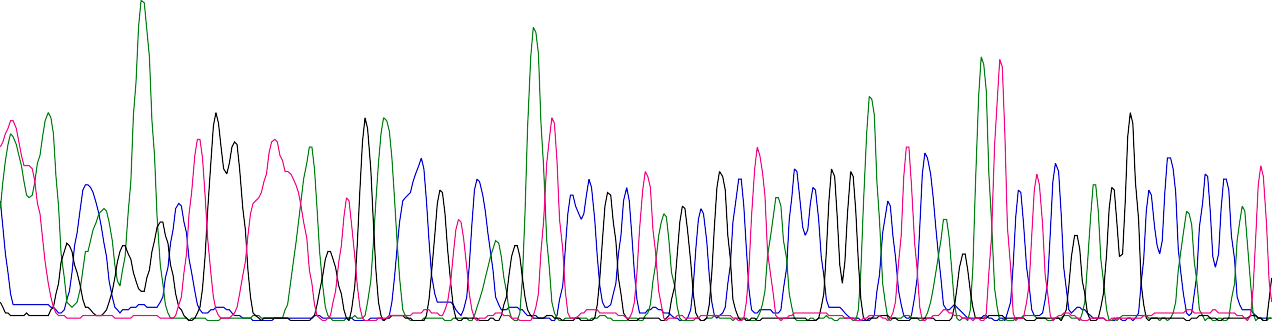


B3837 TRAF6.seq(1>1569)

# ATAGCAGAGCTGGTTTAGTGACCGTCAGATCCGCTAGCGCTACCGGACTCAGATCTCGAGGCCACCAT


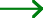
at

70 80 90 100 110 120 130


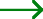
 GAGTCTGCTAAACTGTGAAAACAGCTGTGGATCCAGCCAGTCTGAAAGTGACTGCTGTGTGGCCATGG A2506_1-CMV_F-Z171106087A_G01.ab1(50>600)


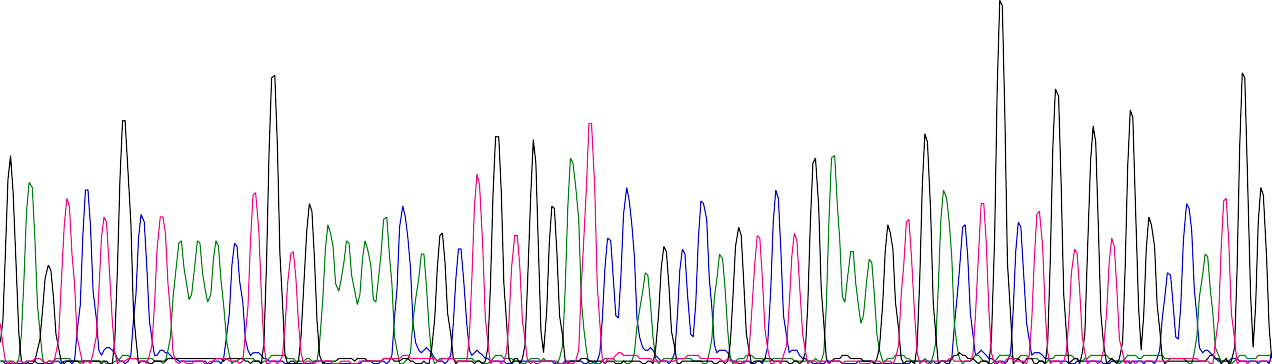


B3837 TRAF6.seq(1>1569)

# GAGTCTGCTAAACTGTGAAAACAGCTGTGGATCCAGCCAGTCTGAAAGTGACTGCTGTGTGGCCATGG


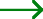
gagtctgctaaactgtgaaaacagctgtggatccagccagtctgaaagtgactgctgtgtggccatgg

140 150 160 170 180 190 200

# CCAGCTCCTGTAGCGCTGTAACAAAAGATGATAGTGTGGGTGGAACTGCCAGCACGGGGAACCTCTCC

140 150 160 170 180 190 200


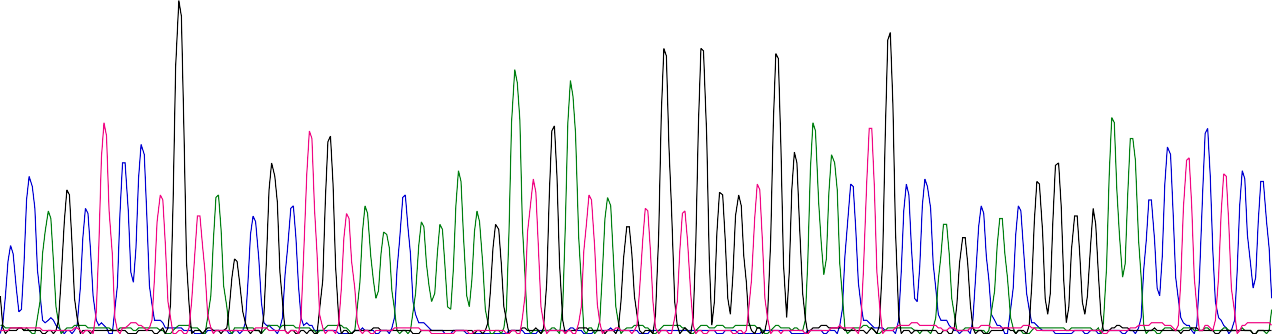

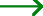
 CCAGCTCCTGTAGCGCTGTAACAAAAGATGATAGTGTGGGTGGAACTGCCAGCACGGGGAACCTCTCC A2506_1-CMV_F-Z171106087A_G01.ab1(50>600)

B3837 TRAF6.seq(1>1569)

# CCAGCTCCTGTAGCGCTGTAACAAAAGATGATAGTGTGGGTGGAACTGCCAGCACGGGGAACCTCTCC


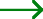
ccagctcctgtagcgctgtaacaaaagatgatagtgtgggtggaactgccagcacggggaacctctcc

210 220 230 240 250 260 270


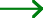
 AGCTCATTTATGGAGGAGATCCAGGGATATGATGTAGAGTTTGACCCACCCCTGGAAAGCAAGTATGA A2506_1-CMV_F-Z171106087A_G01.ab1(50>600)


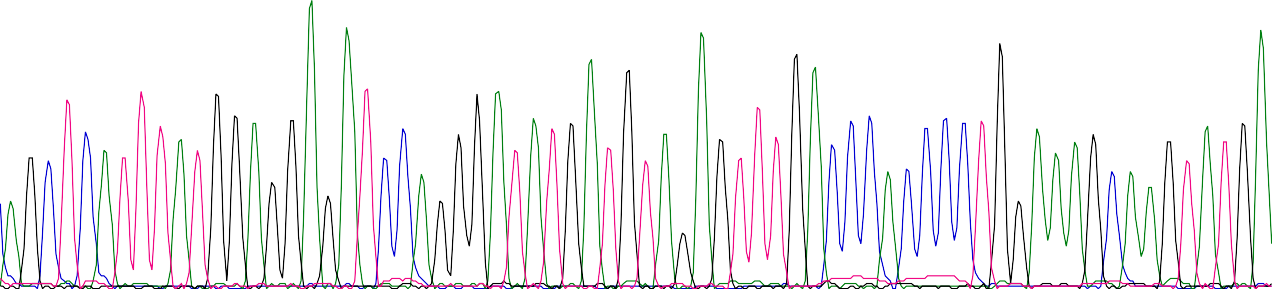


B3837 TRAF6.seq(1>1569)

# AGCTCATTTATGGAGGAGATCCAGGGATATGATGTAGAGTTTGACCCACCCCTGGAAAGCAAGTATGA


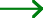
agctcatttatggaggagatccagggatatgatgtagagtttgacccacccctggaaagcaagtatga

280 290 300 310 320 330 340

# ATGCCCCATCTGCTTGATGGCATTACGAGAAGCAGTGCAAACGCCATGCGGCCATAGGTTCTGCAAAG

280 290 300 310 320 330 340


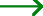
 ATGCCCCATCTGCTTGATGGCATTACGAGAAGCAGTGCAAACGCCATGCGGCCATAGGTTCTGCAAAG A2506_1-CMV_F-Z171106087A_G01.ab1(50>600)


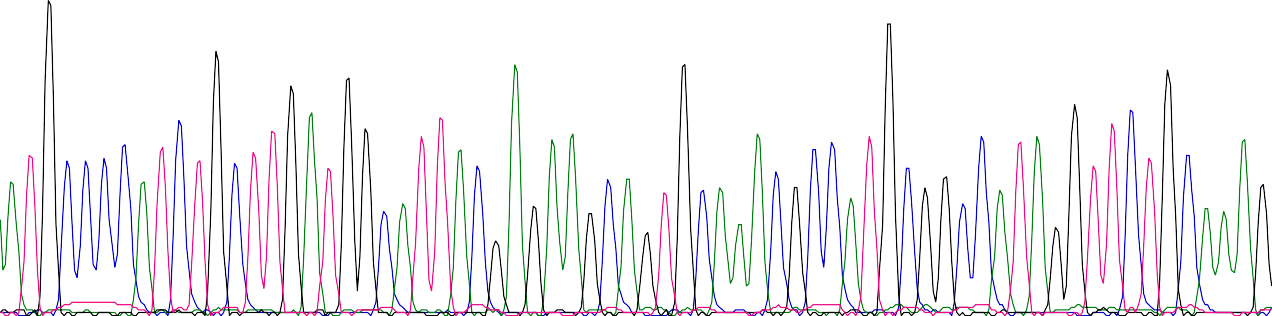


B3837 TRAF6.seq(1>1569)

# ATGCCCCATCTGCTTGATGGCATTACGAGAAGCAGTGCAAACGCCATGCGGCCATAGGTTCTGCAAAG


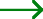
atgccccatctgcttgatggcattacgagaagcagtgcaaacgccatgcggccataggttctgcaaag

350 360 370 380 390 400


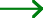
 CCTGCATCATAAAATCAATAAGGGATGCAGGTCACAAATGTCCAGTTGACAATGAAATACTGCTGGAA A2506_1-CMV_F-Z171106087A_G01.ab1(50>600)


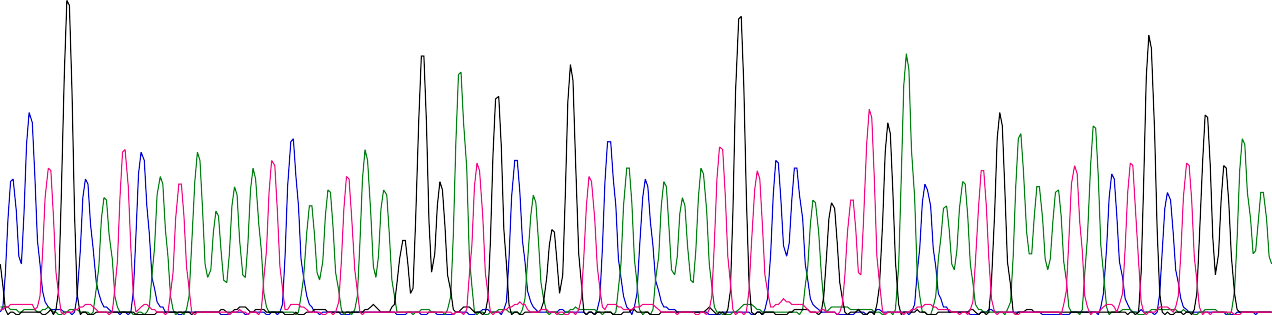


B3837 TRAF6.seq(1>1569)

# CCTGCATCATAAAATCAATAAGGGATGCAGGTCACAAATGTCCAGTTGACAATGAAATACTGCTGGAA


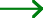
cctgcatcataaaatcaataagggatgcaggtcacaaatgtccagttgacaatgaaatactgctggaa

410 420 430 440 450 460 470

# AATCAACTATTTCCAGACAATTTTGCAAAACGTGAGATTCTTTCTCTGATGGTGAAATGTCCAAATGA

410 420 430 440 450 460 470


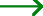
 AATCAACTATTTCCAGACAATTTTGCAAAACGTGAGATTCTTTCTCTGATGGTGAAATGTCCAAATGA A2506_1-CMV_F-Z171106087A_G01.ab1(50>600)


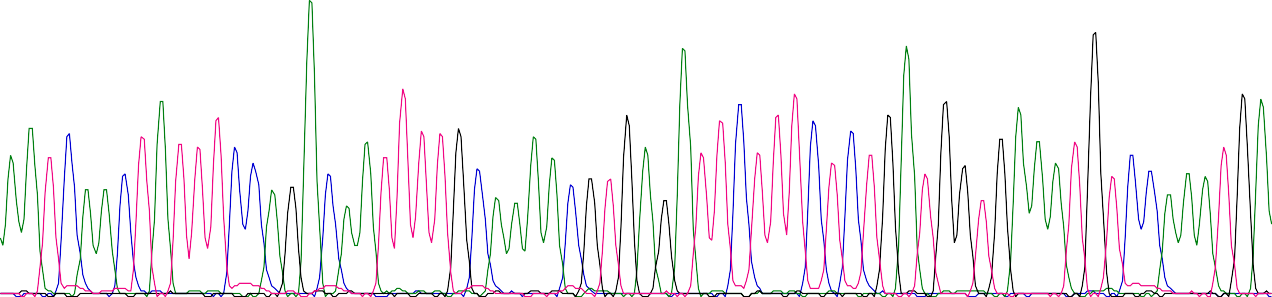


B3837 TRAF6.seq(1>1569)

# AATCAACTATTTCCAGACAATTTTGCAAAACGTGAGATTCTTTCTCTGATGGTGAAATGTCCAAATGA


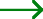
aatcaactatttccagacaattttgcaaaacgtgagattctttctctgatggtgaaatgtccaaatga

480 490 500 510 520 530 540


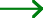
 AGGTTGTTTGCACAAGATGGAACTGAGACATCTTGAGGATCATCAAGCACATTGTGAGTTTGCTCTTA A2506_1-CMV_F-Z171106087A_G01.ab1(50>600)


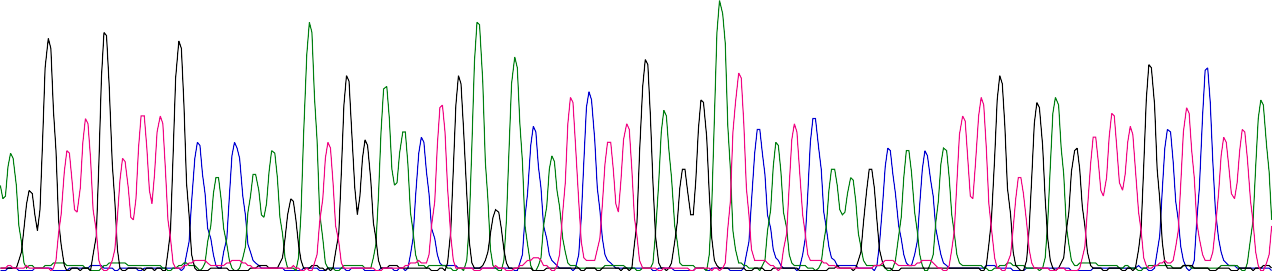


B3837 TRAF6.seq(1>1569)

# AGGTTGTTTGCACAAGATGGAACTGAGACATCTTGAGGATCATCAAGCACATTGTGAGTTTGCTCTTA


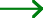
aggttgtttgcacaagatggaactgagacatcttgaggatcatcaagcacattgtgagtttgctctta

550 560 570 580 590 600 610

# TGGATTGTCCCCAATGCCAGCGTCCCTTCCAAAAATTCCATATTAATATTCACATTCTGAAGGATTGT

550 560 570 580 590 600 610


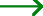
 TGGATTGTCCCCAATGCCAGCGTCCCTTCCAAAAATTCCATATTAATATTCACATTCTGAAGGATTGT A2506_1-CMV_F-Z171106087A_G01.ab1(50>600)


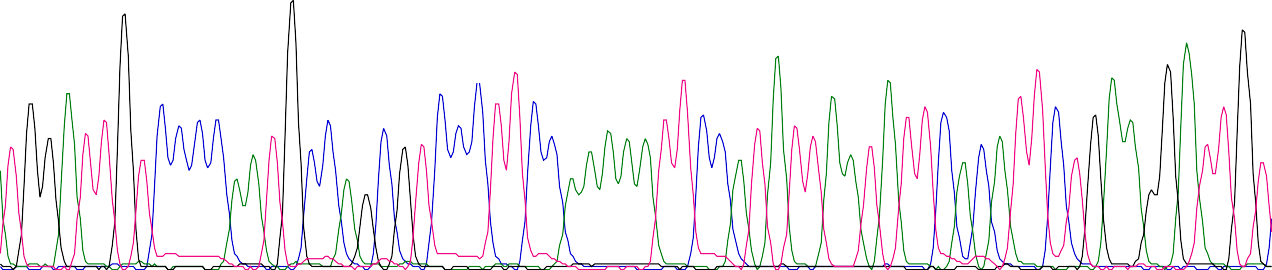


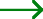
B3837 TRAF6.seq(1>1569)


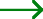
A2506_1-B3837F1-Z171106087A_B11.ab1(50>700)

# TGGATTGTCCCCAATGCCAGCGTCCCTTCCAAAAATTCCATATTAATATTCACATTCTGAAGGATTGT

tggattgtccccaatgccagcgtcccttccaaaaattccatattaatattcacattctgaaggattgt

# TCTGAAGGATTGT

620 630 640 650 660 670 680


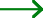
 CCAAGGAGACAGGTTTCTTGTGACAACTGTGCTGCATCAATGGCATTTGAAGATAAAGAGATCCATGA A2506_1-CMV_F-Z171106087A_G01.ab1(50>600)


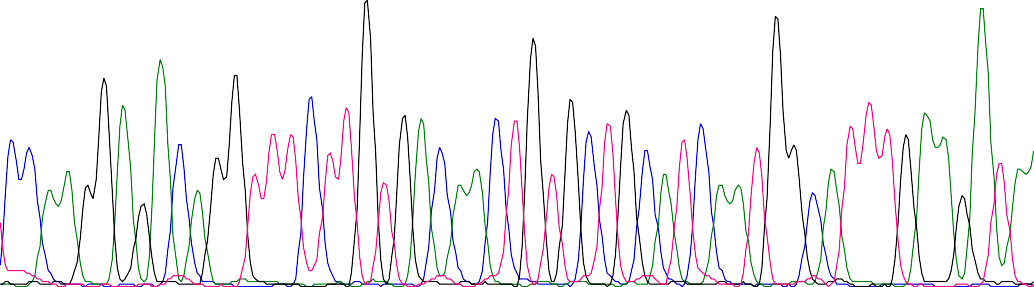


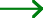
B3837 TRAF6.seq(1>1569)


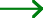
A2506_1-B3837F1-Z171106087A_B11.ab1(50>700)

# CCAAGGAGACAGGTTTCTTGTGACAACTGTGCTGCATCAATGGCATTTGAAGATA

ccaaggagacaggtttcttgtgacaactgtgctgcatcaatggcatttgaagataaagagatccatga


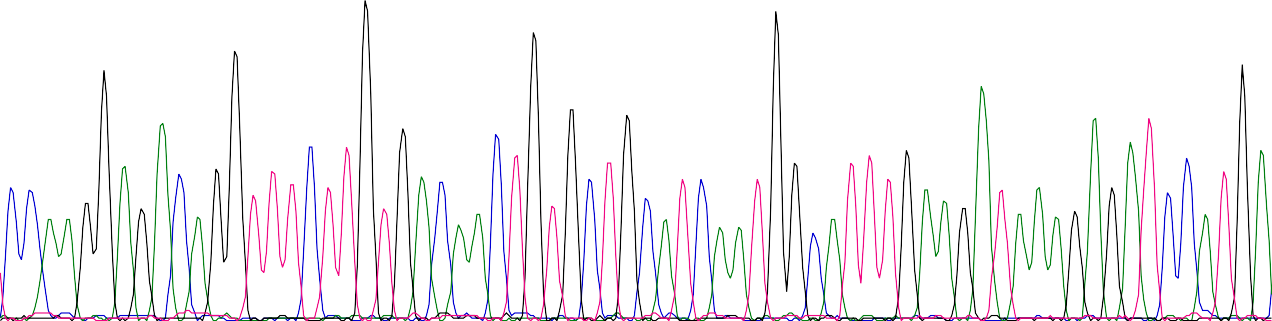


# CCAAGGAGACAGGTTTCTTGTGACAACTGTGCTGCATCAATGGCATTTGAAGATAAAGAGATCCATGA

690 700 710 720 730 740

# CCAGAACTGTCCTTTGGCAAATGTCATCTGTGAATACTGCAATACTATACTCATCAGAGAACAGATGC


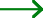
B3837 TRAF6.seq(1>1569)


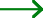
A2506_1-B3837F1-Z171106087A_B11.ab1(50>700)

ccagaactgtcctttggcaaatgtcatctgtgaatactgcaatactatactcatcagagaacagatgc


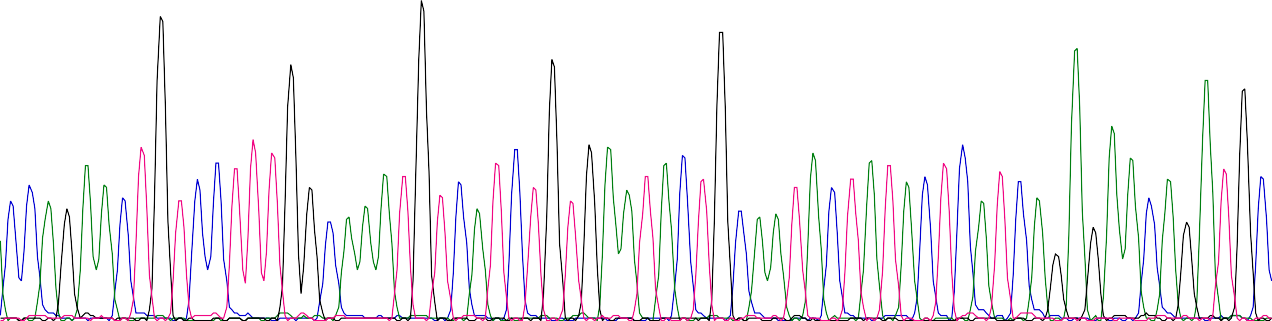


# CCAGAACTGTCCTTTGGCAAATGTCATCTGTGAATACTGCAATACTATACTCATCAGAGAACAGATGC

750 760 770 780 790 800 810

# CTAATCATTATGATCTAGACTGCCCTACAGCCCCAATTCCATGCACATTCAGTACTTTTGGTTGCCAT


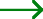
B3837 TRAF6.seq(1>1569)


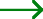
A2506_1-B3837F1-Z171106087A_B11.ab1(50>700)

ctaatcattatgatctagactgccctacagccccaattccatgcacattcagtacttttggttgccat

# CTAATCATTATGATCTAGACTGCCCTACAGCCCCAATTCCATGCACATTCAGTACTTTTGGTTGCCAT


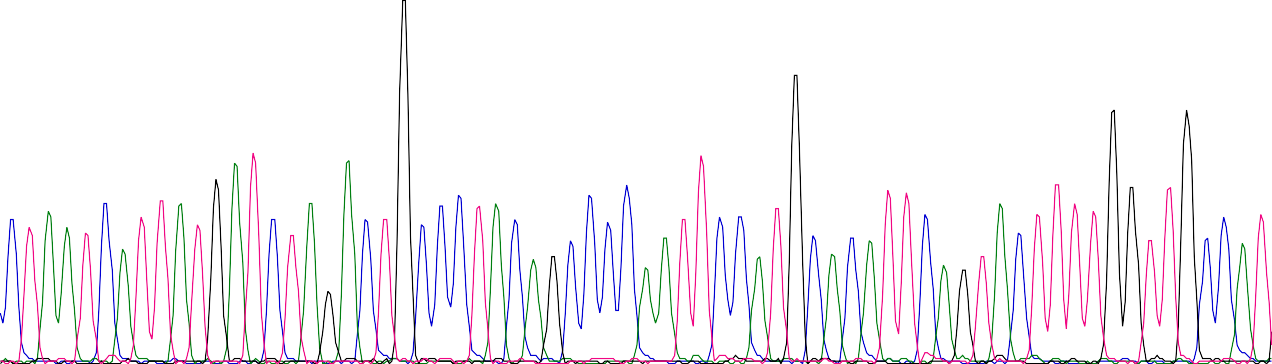


820 830 840 850 860 870 880

# GAAAAGATGCAGAGGAATCACTTGGCACGCCACCTACAAGAGAACACCCAGTCACACATGAGAATGTT


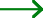
B3837 TRAF6.seq(1>1569)


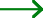
A2506_1-B3837F1-Z171106087A_B11.ab1(50>700)

gaaaagatgcagaggaatcacttggcacgccacctacaagagaacacccagtcacacatgagaatgtt


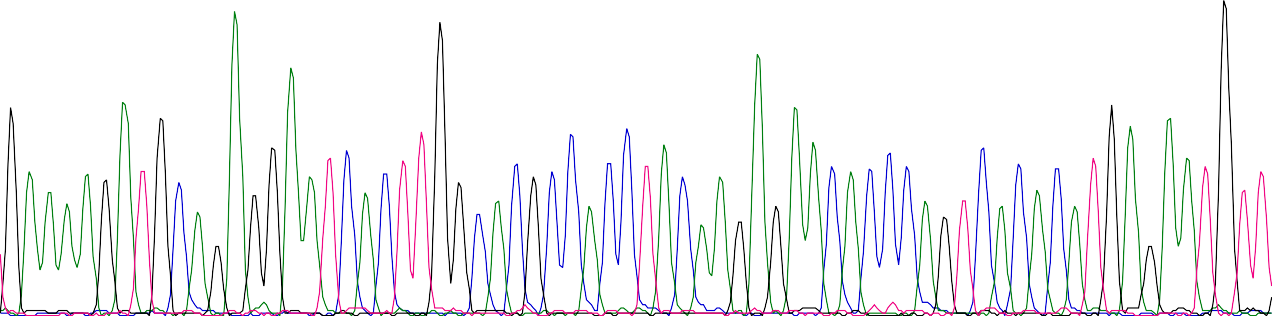


# GAAAAGATGCAGAGGAATCACTTGGCACGCCACCTACAAGAGAACACCCAGTCACACATGAGAATGTT

890 900 910 920 930 940 950

# GGCCCAGGCTGTTCATAGTTTGAGCGTTATACCCGACTCTGGGTATATCTCAGAGGTCCGGAATTTCC


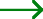
B3837 TRAF6.seq(1>1569)


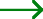
A2506_1-B3837F1-Z171106087A_B11.ab1(50>700)

ggcccaggctgttcatagtttgagcgttatacccgactctgggtatatctcagaggtccggaatttcc


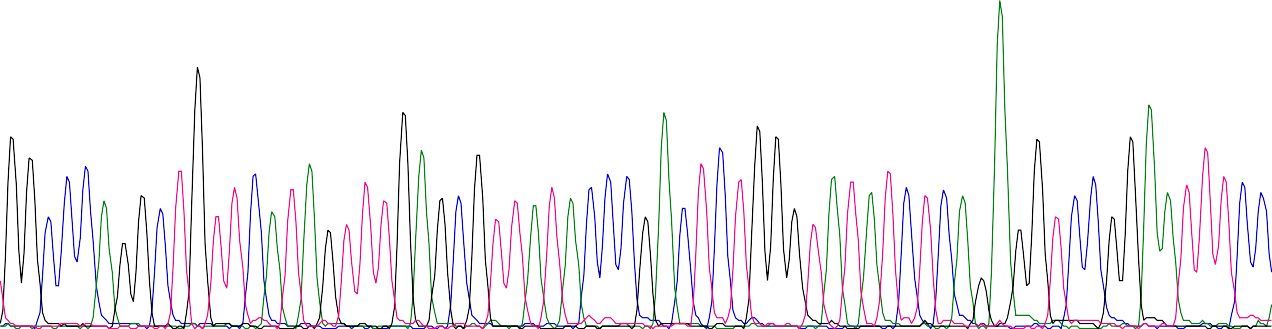


# GGCCCAGGCTGTTCATAGTTTGAGCGTTATACCCGACTCTGGGTATATCTCAGAGGTCCGGAATTTCC

960 970 980 990 1000 1010 1020

# AGGAAACTATTCACCAGTTAGAGGGTCGCCTTGTAAGACAAGACCATCAAATCCGGGAGCTGACTGCT


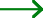
B3837 TRAF6.seq(1>1569)


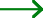
A2506_1-B3837F1-Z171106087A_B11.ab1(50>700)

aggaaactattcaccagttagagggtcgccttgtaagacaagaccatcaaatccgggagctgactgct


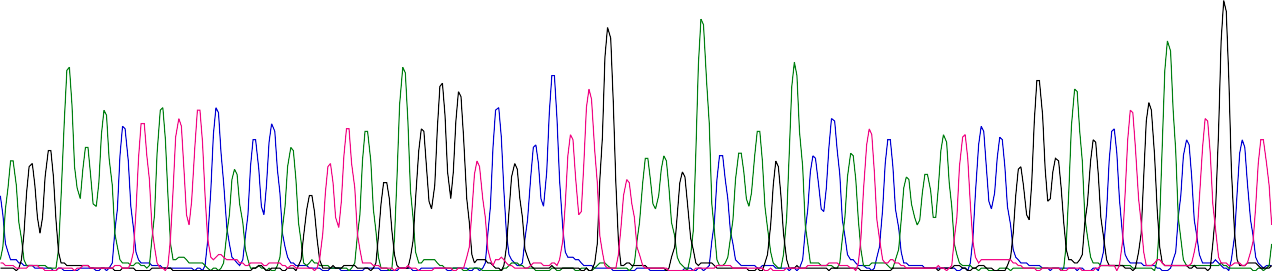


# AGGAAACTATTCACCAGTTAGAGGGTCGCCTTGTAAGACAAGACCATCAAATCCGGGAGCTGACTGCT

1030 1040 1050 1060 1070 1080

# AAAATGGAAACTCAGAGTATGTATGTAAGTGAGCTCAAACGAACCATTCGAACCCTTGAGGACAAAGT


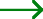
B3837 TRAF6.seq(1>1569)


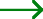
A2506_1-B3837F1-Z171106087A_B11.ab1(50>700)

aaaatggaaactcagagtatgtatgtaagtgagctcaaacgaaccattcgaacccttgaggacaaagt


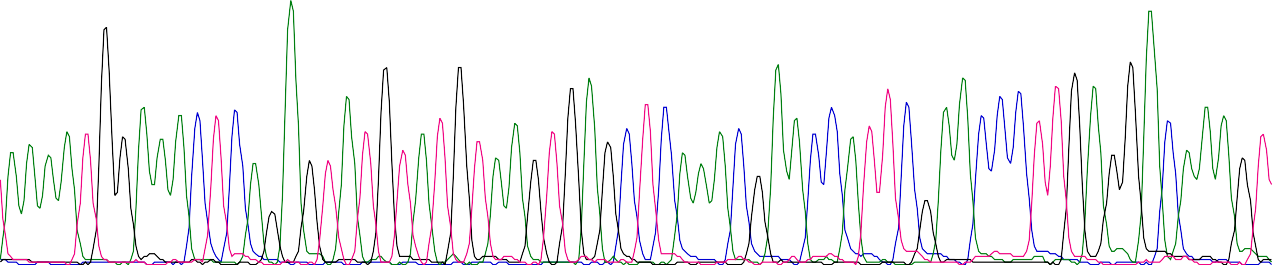


# AAAATGGAAACTCAGAGTATGTATGTAAGTGAGCTCAAACGAACCATTCGAACCCTTGAGGACAAAGT

1090 1100 1110 1120 1130 1140 1150

# TGCTGAAATCGAAGCACAGCAGTGCAATGGAATTTATATTTGGAAGATTGGCAACTTTGGAATGCATT


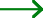
B3837 TRAF6.seq(1>1569)


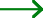
A2506_1-B3837F1-Z171106087A_B11.ab1(50>700)


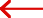
A2506_1-PEX_3R-Z171106087A_C11.ab1(50>700)

tgctgaaatcgaagcacagcagtgcaatggaatttatatttggaagattggcaactttggaatgcatt


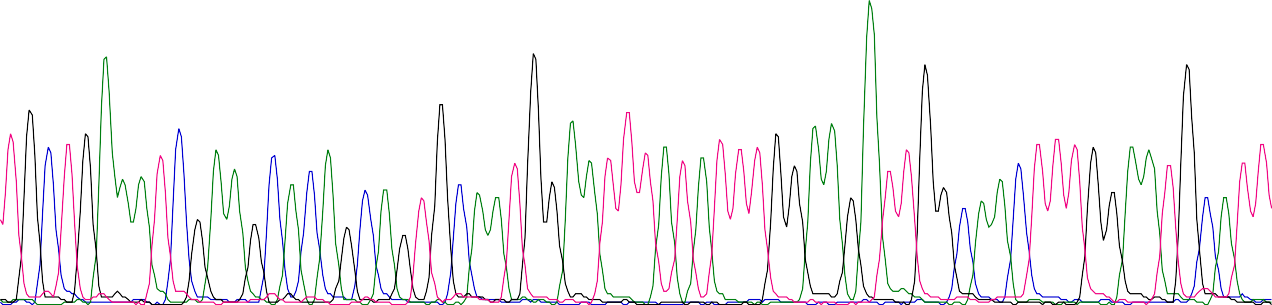


# TGCTGAAATCGAAGCACAGCAGTGCAATGGAATTTATATTTGGAAGATTGGCAACTTTGGAATGCATT


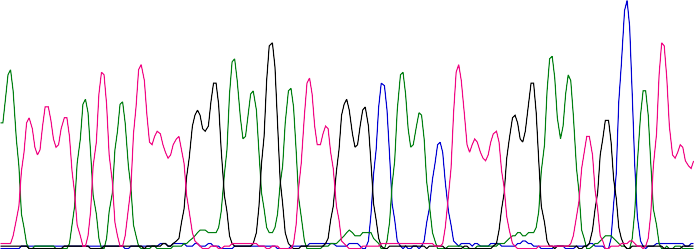


ATTTATATTTGGAAGATTGGCAACTTTGGAATGCATT

1160 1170 1180 1190 1200 1210 1220

# TGAAATGTCAAGAAGAGGAGAAACCTGTTGTGATTCATAGCCCTGGATTCTACACTGGCAAACCCGGG


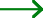
B3837 TRAF6.seq(1>1569)


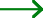
A2506_1-B3837F1-Z171106087A_B11.ab1(50>700)


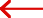
A2506_1-PEX_3R-Z171106087A_C11.ab1(50>700)

tgaaatgtcaagaagaggagaaacctgttgtgattcatagccctggattctacactggcaaacccggg


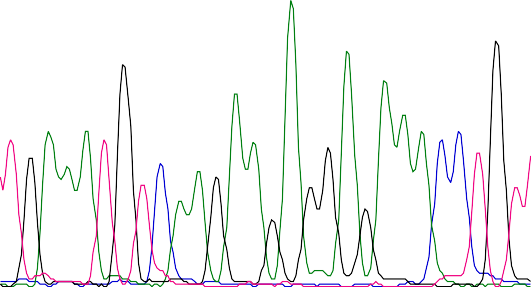


# TGAAATGTCAAGAAGAGGAGAAACCTGT


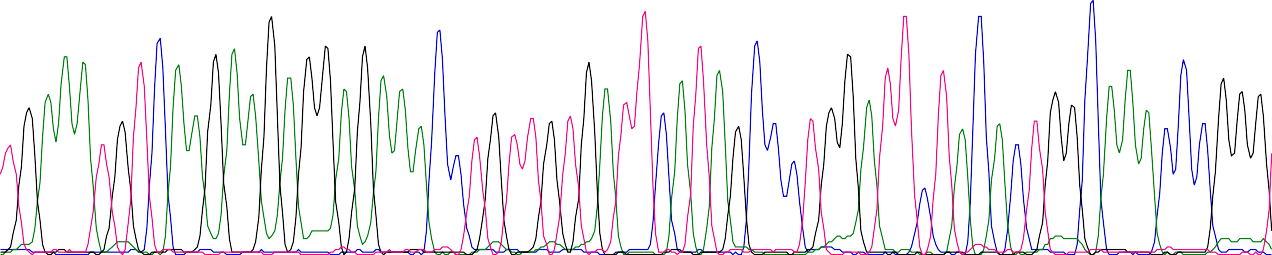


TGAAATGTCAAGAAGAGGAGAAACCTGTTGTGATTCATAGCCCTGGATTCTACACTGGCAAACCCGGG

1230 1240 1250 1260 1270 1280 1290

#
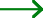
 TACAAACTGTGCATGCGCTTGCACCTTCAGTTACCGACTGCTCAGCGCTGTGCAAACTATATATCCCT

B3837 TRAF6.seq(1>1569)

A2506_1-PEX_3R-Z171106087A_C11.ab1(50>700)

tacaaactgtgcatgcgcttgcaccttcagttaccgactgctcagcgctgtgcaaactatatatccct


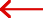


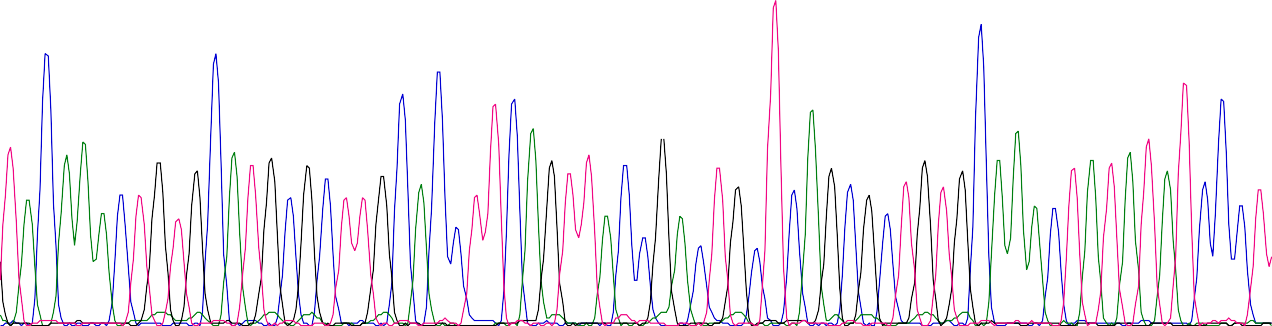


# TACAAACTGTGCATGCGCTTGCACCTTCAGTTACCGACTGCTCAGCGCTGTGCAAACTATATATCCCT

1300 1310 1320 1330 1340 1350 1360

#
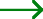
 TTTTGTCCACACAATGCAAGGAGAATATGACAGCCACCTCCCTTGGCCCTTCCAGGGTACAATACGCC

B3837 TRAF6.seq(1>1569)

A2506_1-PEX_3R-Z171106087A_C11.ab1(50>700)

ttttgtccacacaatgcaaggagaatatgacagccacctcccttggcccttccagggtacaatacgcc


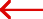


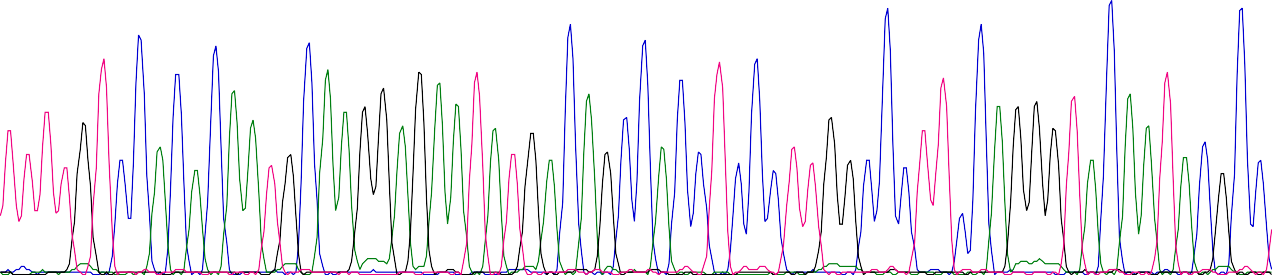


# TTTTGTCCACACAATGCAAGGAGAATATGACAGCCACCTCCCTTGGCCCTTCCAGGGTACAATACGCC

1370 1380 1390 1400 1410 1420

# TTACAATTCTTGATCAGTCTGAAGCACCTGTAAGGCAAAACCACGAAGAGATAATGGATGCCAAACCA

B3837 TRAF6.seq(1>1569)

A2506_1-PEX_3R-Z171106087A_C11.ab1(50>700)

ttacaattcttgatcagtctgaagcacctgtaaggcaaaaccacgaagagataatggatgccaaacca


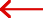

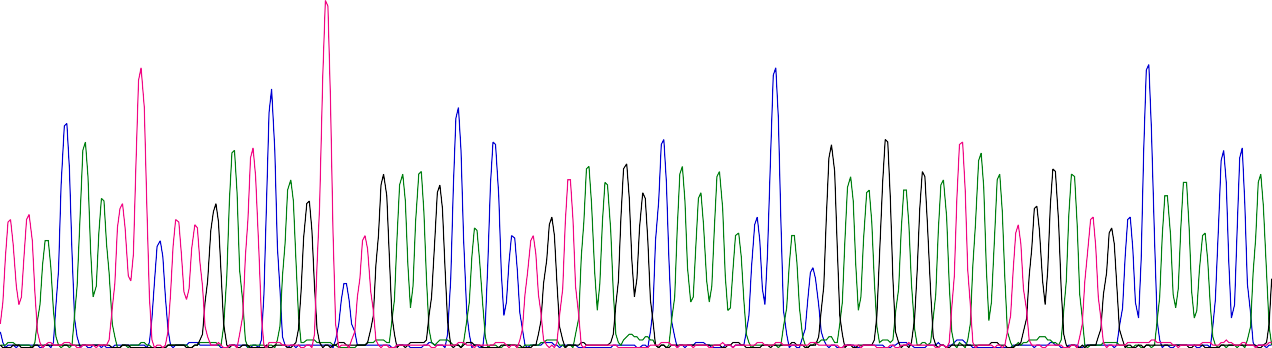


#
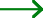
TTACAATTCTTGATCAGTCTGAAGCACCTGTAAGGCAAAACCACGAAGAGATAATGGATGCCAAACCA

1430 1440 1450 1460 1470 1480 1490

#
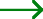
 GAGCTGCTTGCTTTCCAGCGACCCACAATCCCACGGAACCCAAAAGGTTTTGGCTATGTAACTTTTAT

B3837 TRAF6.seq(1>1569)

A2506_1-PEX_3R-Z171106087A_C11.ab1(50>700)

gagctgcttgctttccagcgacccacaatcccacggaacccaaaaggttttggctatgtaacttttat


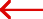


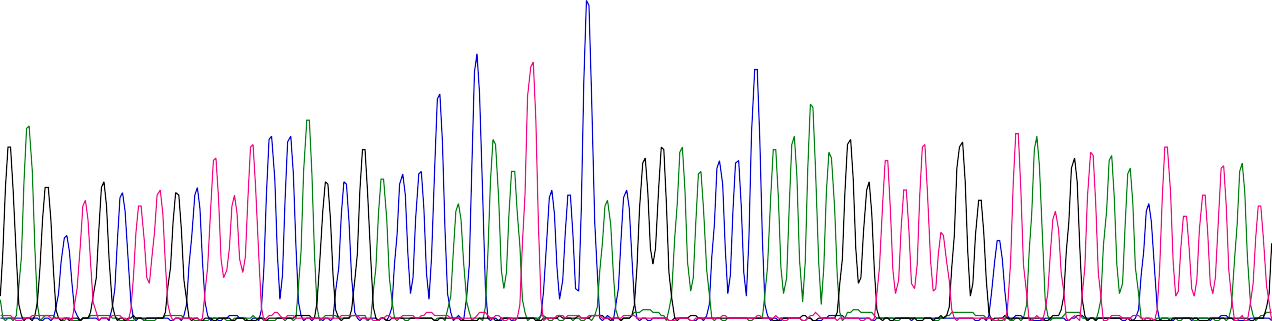


# GAGCTGCTTGCTTTCCAGCGACCCACAATCCCACGGAACCCAAAAGGTTTTGGCTATGTAACTTTTAT

1500 1510 1520 1530 1540 1550 1560

#
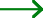
 GCATCTGGAAGCCCTAAGACAAAGAACTTTCATTAAGGATGACACATTATTAGTGCGCTGTGAGGTCT

B3837 TRAF6.seq(1>1569)

A2506_1-PEX_3R-Z171106087A_C11.ab1(50>700)

gcatctggaagccctaagacaaagaactttcattaaggatgacacattattagtgcgctgtgaggtct


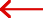


# GCATCTGGAAGCCCTAAGACAAAGAACTTTCATTAAGGATGACACATTATTAGTGCGCTGTGAGGTCT


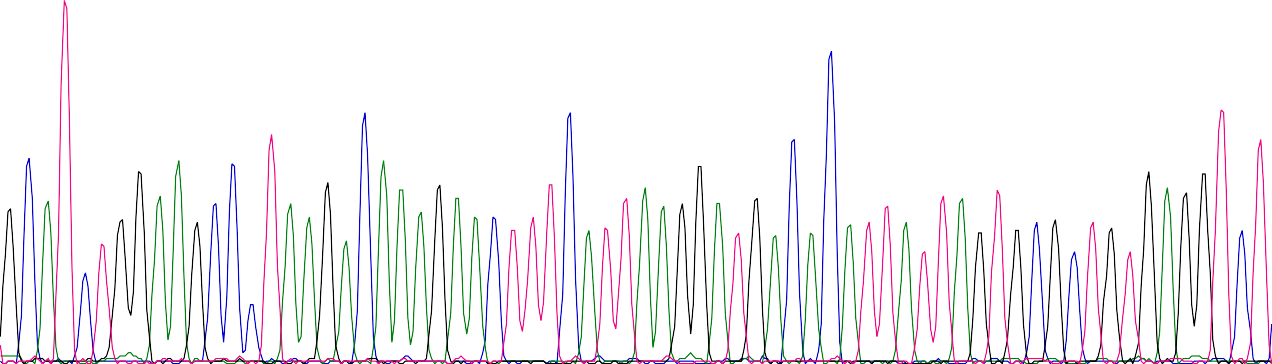


1570 1580 1590 1600 1610 1620 1630

#
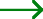
 CCACCCGCTTTGACATGGGTAGCCTTCGGAGGGAGGGTTTTCAGCCACGAAGTACTGATGCAGGGGTA

B3837 TRAF6.seq(1>1569)

A2506_1-PEX_3R-Z171106087A_C11.ab1(50>700)

ccacccgctttgacatgggtagccttcggagggagggttttcagccacgaagtactgatgcaggggta


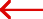


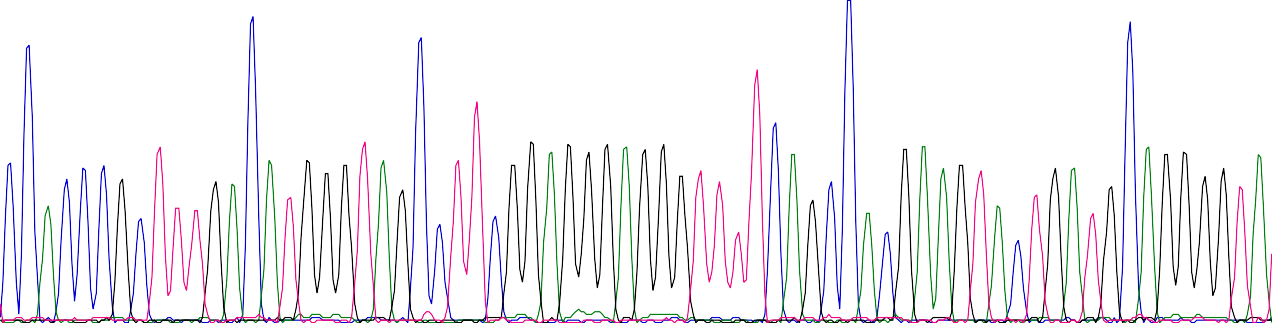


# CCACCCGCTTTGACATGGGTAGCCTTCGGAGGGAGGGTTTTCAGCCACGAAGTACTGATGCAGGGGTA

1640 1650 1660 1670 1680 1690 1700

# TAGGAATTCTGCAGTCGACGGTACCGCGGGCCCGGGATCCATCACCGGTATCGGCGCGCCATTGATAT

B3837 TRAF6.seq(1>1569)

A2506_1-PEX_3R-Z171106087A_C11.ab1(50>700)

tag


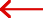

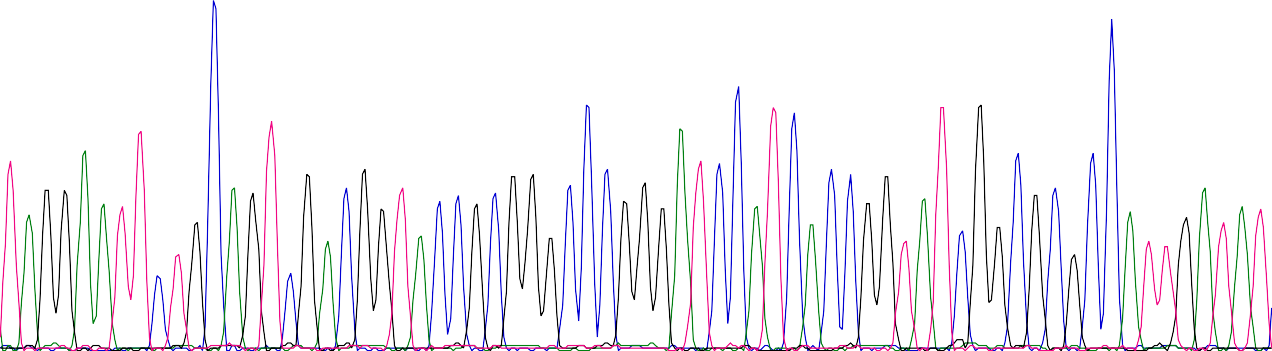


#
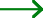
TAGGAATTCTGCAGTCGACGGTACCGCGGGCCCGGGATCCATCACCGGTATCGGCGCGCCATTGATAT

1710


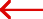
 CA A2506_1-PEX_3R-Z171106087A_C11.ab1(50>700)

CA
